# A LAT1-selective PET tracer, 5-[¹⁸F]F-αMe-3BPA, as a companion to its structurally matched ¹⁰B analog in boron neutron capture therapy

**DOI:** 10.1007/s00259-025-07668-3

**Published:** 2025-11-18

**Authors:** Naoya Kondo, Fuko Hirano, Yasukazu Kanai, Kensuke Suzuki, Anna Miyazaki, Takashi Temma

**Affiliations:** 1 Department of Biofunctional Analysis, Graduate School of Pharmaceutical Sciences, Osaka Medical and Pharmaceutical University, 4-20-1 Nasahara, Takatsuki, 569-1094 Osaka Japan; 2 Division of Fundamental Technology Development, Near InfraRed Photo-ImmunoTherapy Research Institute, Kansai Medical University, 2-5-1 Shin-machi, Hirakata, 573-1010 Japan; 3 Kansai BNCT Medical Center, Educational Foundation of Osaka Medical and Pharmaceutical University, 2-7 Daigakumachi, Takatsuki, 569-8686 Osaka Japan; 4 Stella Pharma Corporation Sakai R&D Center, Bldg. C-23, 1-1 Gakuen-cho, Naka-ku, Sakai, 599-8531 Osaka Japan; 5 Particle Radiation Biology, Institute for Integrated Radiation and Nuclear Science, Kyoto University, 2-1010 Asashiro Nishi, Kumatori, Sennan-gun, 590-0494 Osaka Japan

**Keywords:** Boron neutron capture therapy, 4-borono-L-phenylalanine, PET, Copper-catalyzed radiofluorination, Theranostics, LAT1

## Abstract

**Purpose:**

This study aimed to develop and evaluate 5-[¹⁸F]F-αMe-3BPA, a novel PET probe designed as a theranostic partner for 5F-αMe-3[¹⁰B]BPA in Boron Neutron Capture Therapy (BNCT). The goal was to address limitations of the clinically used BPA/[¹⁸F]FBPA pair, including poor water solubility, limited LAT1 specificity, and suboptimal diagnostic performance, thereby improving tumor-selective drug delivery and enabling accurate prediction of therapeutic biodistribution through structure-matched PET/BNCT.

**Methods:**

LAT1 dependency was tested in vitro using cancer cell lines with differential LAT1 expression. In vivo biodistribution of both therapeutic agents and their ¹⁸F-labeled analogs were assessed in xenograft mouse models. Radiosynthesis of 5-[¹⁸F]F-αMe-3BPA was achieved via copper-catalyzed nucleophilic radiofluorination. PET/CT imaging compared tumor visualization with [¹⁸F]FBPA. Co-injection studies (5-[¹⁸F]F-αMe-3BPA/5F-αMe-3BPA and [¹⁸F]FBPA/BPA) quantitatively evaluated concordance between ¹⁸F radioactivity and boron concentrations across tissues.

**Results:**

5F-αMe-3BPA uptake in cancer cells was strictly LAT1-dependent. In LAT1-high T3M-4 xenografts, its tumor-to-muscle boron ratio (22) far exceeded BPA (3.4). 5-[¹⁸F]F-αMe-3BPA was synthesized successfully and achieved a tumor-to-muscle ratio of 29 versus 5.3 for [¹⁸F]FBPA. PET imaging showed clear, high-contrast visualization of T3M-4 tumors, with co-injection confirming strong concordance between PET probe and therapeutic agent biodistribution.

**Conclusion:**

The 5-[¹⁸F]F-αMe-3BPA/5F-αMe-3[¹⁰B]BPA theranostic pair demonstrates high LAT1 specificity, low normal tissue uptake, and strong pharmacokinetic alignment, enabling accurate prediction of therapeutic boron delivery for BNCT. Furthermore, 5-[¹⁸F]F-αMe-3BPA shows promise as a dedicated LAT1 imaging probe for advancing LAT1-targeted therapies.

**Supplementary Information:**

The online version contains supplementary material available at 10.1007/s00259-025-07668-3.

## Introduction

Boron Neutron Capture Therapy (BNCT) is a targeted radiotherapy that harnesses the nuclear reaction between boron-10 (¹⁰B) and externally applied epithermal neutrons. This reaction generates high–linear energy transfer particles—an α-particle and a recoiling ^7^Li nucleus—each with a penetration range of less than one cell diameter (~ 10 μm). When ¹⁰B is selectively delivered into tumor cells, BNCT enables the localized destruction of malignant cells while sparing adjacent healthy tissue, thereby achieving cell-level therapeutic precision [1–3]. Unlike conventional radionuclide therapies, which continuously emit radiation according to systemic biodistribution, BNCT represents an “off–on” strategy. Radiolabeled probes, including targeted alpha therapies (TAT), remain “always-on,” exposing normal tissues to background radiation during circulation. In contrast, BNCT decouples drug delivery from radiation activation: boron carriers are pharmacologically inert until triggered by external neutron irradiation [4]. This switchable mechanism provides both temporal and spatial control of therapeutic action, minimizing off-target toxicity and expanding the therapeutic window. The dosimetric framework of BNCT differs fundamentally from systemic radionuclide therapies. In TAT, dose optimization focuses on systemic exposure, with safety determined by off-target deposition in radiosensitive organs such as the kidneys and bone marrow, where unintended hematologic or renal toxicities may arise. In contrast, BNCT confines radiation delivery to tissues within the neutron field, making the microdistribution of ¹⁰B the critical determinant of safety. Clinical risk is driven by boron uptake in normal tissues adjacent to tumors—such as muscle, skin, or brain parenchyma—particularly relevant in head and neck cancer applications [5]. Thus, boron carriers must provide high tumor-to-normal tissue contrast rather than relying solely on tumor uptake. The clinically approved agent, 4-borono-L-phenylalanine (BPA), is transported into tumor cells via L-type amino acid transporter 1 (LAT1) [6], which is highly expressed in many types of cancers [7, 8]. 4-Borono-2-[¹⁸F]fluoro-L-phenylalanine [¹⁸F]FBPA) is tested to estimate the BPA biodistribution by PET [9]. However, both compounds are also LAT2 substrates, which are broadly expressed in normal tissues [6, 10], resulting in modest tumor-to-muscle (T/M) ratios (~ 4) [11, 12]. While no significant differences have been reported in the biodistribution of BPA and [^19^F]FBPA following subcutaneous administration [11], the presence of fluorine in [¹⁸F]FBPA results in structural non-identity with BPA, indicating that ideal theranostic pairing remains to be fully achieved.

**Fig. 1:**
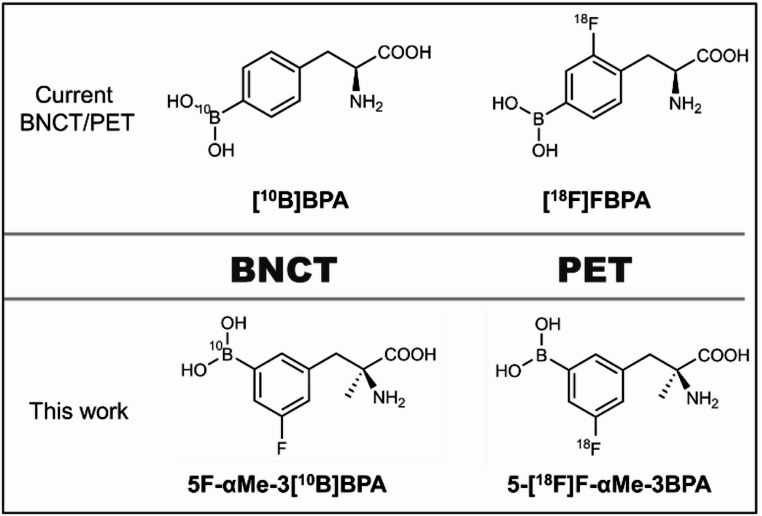
Comparison of current and novel BNCT/PET theranostic approaches

We previously identified 3-borono-5-fluoro-L-α-methylphenylalanine (5F-αMe-3BPA) as a novel fluorinated boron compound with high water solubility, strong LAT1 selectivity, and significantly improved T/M ratios compared with BPA in tumor-bearing mice [13, 14]. Building on this foundation, the present study sought to develop 5-[¹⁸F]F-αMe-3BPA as a theranostic counterpart to 5F-αMe-3[¹⁰B]BPA, thereby enabling precise PET/BNCT integration (Fig. 1). We synthesized 5-[¹⁸F]F-αMe-3BPA via copper-catalyzed radiofluorination and systematically assessed its in vitro and in vivo performance. The study objectives included confirming LAT1-dependent uptake, validating in vivo co-localization of natural boron and ¹⁸F through co-administration, and evaluating tumor selectivity and imaging performance relative to [¹⁸F]FBPA. Collectively, these results establish 5-[¹⁸F]F-αMe-3BPA as a highly LAT1-specific, pharmacokinetically concordant PET tracer with strong potential for accurate patient selection and treatment planning in BNCT using 5F-αMe-3[¹⁰B]BPA.

## Materials and methods

### General

All reagents were purchased from Fujifilm Wako Pure Chemical Corporation (Osaka, Japan), Tokyo Chemical Industry Co., Ltd. (Tokyo, Japan), or Nacalai Tesque (Kyoto, Japan) and were used without additional purification. ^1^H and ^13^C NMR spectra were recorded on a JNM-ECZ400S spectrometer (JEOL Ltd., Tokyo, Japan). High-resolution mass spectrometry was performed on a JMS-700(2) instrument (JEOL Ltd.).

### Cell lines

Human pancreatic cancer T3M-4 cells were obtained from the RIKEN BioResource Research Center (Ibaraki, Japan). Human lung cancer A549 cells were purchased from the American Type Culture Collection (Manassas, VA, USA), and human glioblastoma U-87 MG cells were kindly provided by Dr. Magata (Hamamatsu University School of Medicine, Japan). T3M-4 cells were maintained in RPMI-1640 medium supplemented with 10% fetal bovine serum (FBS) and 1% penicillin–streptomycin. A549 and U-87 MG cells were cultured in high-glucose DMEM containing 10% FBS and 1% penicillin–streptomycin. All cell lines were incubated at 37 °C in a humidified atmosphere with 5% CO₂.

### Cellular uptake study

Transporter expression in T3M-4, A549, and U-87 MG cells was assessed by Western blotting (see Supplemental Information, SI). Cellular uptake assays were performed following our previously reported protocol [13, 14] (details in SI). Briefly, cells were seeded in 12-well plates and maintained in Hank’s balanced salt solution (HBSS, pH 7.4). After pre-incubation with HBSS (450 µL) with or without transporter inhibitors (10 µM JPH203 or 1 mM BCH) at 37 °C for 5 min, 50 µL of 1 mM 5F-αMe-3BPA or BPA complexed with D-fructose (0.11% w/v) was added to achieve a final concentration of 100 µM, followed by incubation for 30 min at 37 °C. Cells were then washed and lysed with 0.2 M NaOH. Protein concentrations were quantified using BCA assay kits (Thermo-Fisher Scientific, Tokyo, Japan). For boron measurements, cell lysates were ashed with nitric acid, and boron content was analyzed by inductively coupled plasma mass spectrometry (ICP-MS, Agilent, Santa Clara, CA, USA). Boron accumulation was expressed as the percentage of administered boron per mg of cellular protein (%dose(B)/mg protein).

### Animal preparation

Male BALB/cSlc-nu/nu mice (4 weeks old, *n* = 86) were obtained from Japan SLC Inc. (Shizuoka, Japan) and maintained under a 12-h light/dark cycle with ad libitum access to food and water. For tumor inoculation, T3M-4 cells (2.5 × 10⁶), A549 cells (1.5 × 10⁶), or U-87 MG cells (3 × 10⁶) suspended in 100 µL of medium were subcutaneously injected into the right hind leg under isoflurane anesthesia to minimize discomfort. For PET imaging studies, T3M-4 and U-87 MG cells were injected into the left and right shoulders, respectively. Imaging was conducted following intraperitoneal administration of a mixed anesthetic containing 0.3 mg/kg medetomidine, 4.0 mg/kg midazolam, and 5.0 mg/kg butorphanol. Mice were randomized according to tumor size (> 100 mm²). All animal experiments were performed in compliance with institutional guidelines and were approved by the institutional animal care and use committee (Permission Number: AP23-004).

### In vivo biodistribution (boron accumulation)

LAT1 expression in excised tumors was assessed by immunohistochemistry (details in SI). Tumor-bearing mice received 100 µL of 50 mM 5F-αMe-3BPA or BPA–fructose complex (2.2% w/v) via tail vein injection. At 60 min post-injection, mice were euthanized, and tumors along with selected organs were harvested and weighed. Samples were then ashed, and boron concentration was determined using ICP-MS. Boron accumulation in each tissue was expressed as the percentage of injected dose per gram (%ID(B)/g).

### Radiosynthesis of 5-[¹⁸F]F-αMe-3BPA

**Scheme 1:**
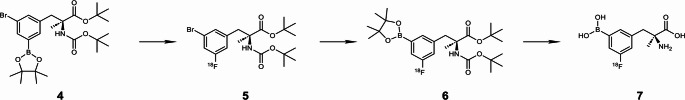
Radiosynthesis of 5-[^18^F]F-αMe-3BPA

Synthetic method of precursor **4** is described in SI. 5F-[¹⁸F]F-αMe-3BPA was synthesized in three steps from precursor **4** (Scheme 1).

- *Step 1.* [¹⁸F]Fluoride was produced via the ¹⁸O(p, n)¹⁸F nuclear reaction using a 20 MeV in-house cyclotron (CYPRIS HM-20, Sumitomo Heavy Industries, Tokyo, Japan). The product (molar activity: 1200 GBq/µmol) was trapped on a Sep-Pak Accell Plus QMA Carbonate Plus Light Cartridge (Waters, MA, USA), eluted with Kryptofix 222 (9.8 mg, 26.0 µmol) and K₂CO₃ (1.8 mg, 13.0 µmol) in acetonitrile/water (4:1, 1 mL), and dried by azeotropic distillation in acetonitrile. The residue was dissolved in n-BuOH/DMA (1:2, 1 mL) and reacted with precursor **4** (5.0 mg, 9.3 µmol) and [Cu(OTf)₂(py)₄] (31.5 mg, 46.5 µmol) at 100 °C for 15 min. Radiochemical conversion was analyzed by TLC (see SI).
- *Step 2.* The eluate (crude **5**) was treated with Pd(dba)₂ (7.4 mg, 12.8 µmol), tricyclohexylphosphine (6.9 mg, 24.5 µmol), bis(pinacolato)diboron (44 mg, 175 µmol), and KOAc (14 mg, 140 µmol), followed by stirring at 120 °C for 30 min. The solution was diluted with water, passed through a Sep-Pak C18 cartridge (Waters), eluted with acetonitrile, and concentrated under reduced pressure.
- *Step 3.* The residue (crude **6**) was treated with acetonitrile (0.4 mL) and HBr (0.35 mL) at 100 °C for 10 min. The final product, 5-[¹⁸F]F-αMe-3BPA (**7**), was purified by RP-HPLC (COSMOSIL 5C18-AR-II, 10 × 250 mm; Nacalai Tesque) using water/acetonitrile (96:4) at 5.0 mL/min (Fig. S1). Radiochemical purity was confirmed by TLC with n-BuOH/water/acetic acid (12:5:3) as the mobile phase.

### Preparation of [¹⁸F]FBPA

[¹⁸F]FBPA was synthesized from 4-borono-L-phenylalanine using [¹⁸F]F₂ on an MPS-200 module (Sumitomo Heavy Industries), following a previously reported method [15]. The product had a radiochemical purity >95% and a molar activity of ~ 100 MBq/µmol.

### In vivo biodistribution of radiotracers

Tumor-bearing mice (*n* = 4 per group) received intravenous injections of 5-[¹⁸F]F-αMe-3BPA (100–370 kBq/100 µL) or [¹⁸F]FBPA (470 kBq/100 µL). At 60 min post-injection, mice were euthanized, and tumors and organs of interest were collected. Organ weight and radioactivity were measured, and results were expressed as percentage of injected dose per gram (%ID(RA)/g). Urinary metabolites of 5-[¹⁸F]F-αMe-3BPA were analyzed by radio-TLC (see SI).

### Co-injection study

The dose of BNCT agents was set at 5 µmol as a practical high-dose condition. T3M-4 tumor-bearing mice (*n* = 4 per group) were injected with either 50 mM 5F-αMe-3BPA plus 5-[¹⁸F]F-αMe-3BPA (370 kBq/100 µL) or 50 mM BPA–fructose (2.2% w/v) plus [¹⁸F]FBPA (400 kBq/100 µL). At 60 min post-injection, organs were excised for weight and radioactivity measurements. %ID(RA)/g was calculated. After radioactivity measurement, samples were ashed, and boron content was measured by ICP-MS. Boron accumulation was calculated as %ID(B)/g.

### PET imaging

Mice bearing T3M-4 and U-87 MG tumors in opposite shoulders (*n* = 4 per group) were injected with 5-[¹⁸F]F-αMe-3BPA (2.5–3.0 MBq/100 µL) or [¹⁸F]FBPA (4.3–5.2 MBq/100 µL). Sequential PET/CT scans were acquired beginning 30 min post-injection on an E-Class VECTor6 CT scanner (MILabs, Houten, Netherlands). PET acquisitions consisted of four 15-min frames, reconstructed with the simultaneous row-action maximum likelihood algorithm (128 subsets, 7 iterations). Tumor regions were manually delineated using Amide software (v1.0.6) to calculate mean SUV values. Maximum intensity projection (MIP) images were generated with Carimas (v2.10) [16]. One necrotic T3M-4 tumor identified on [¹⁸F]FBPA PET was excluded from analysis.

### Statistical analysis

Data are presented as mean ± SD. Statistical tests were performed using GraphPad Prism 8, with test details provided in figure legends or subsections. *P*-values < 0.05 were used to denote statistical significance.

## Results

### Cellular uptake study

**Fig. 2:**
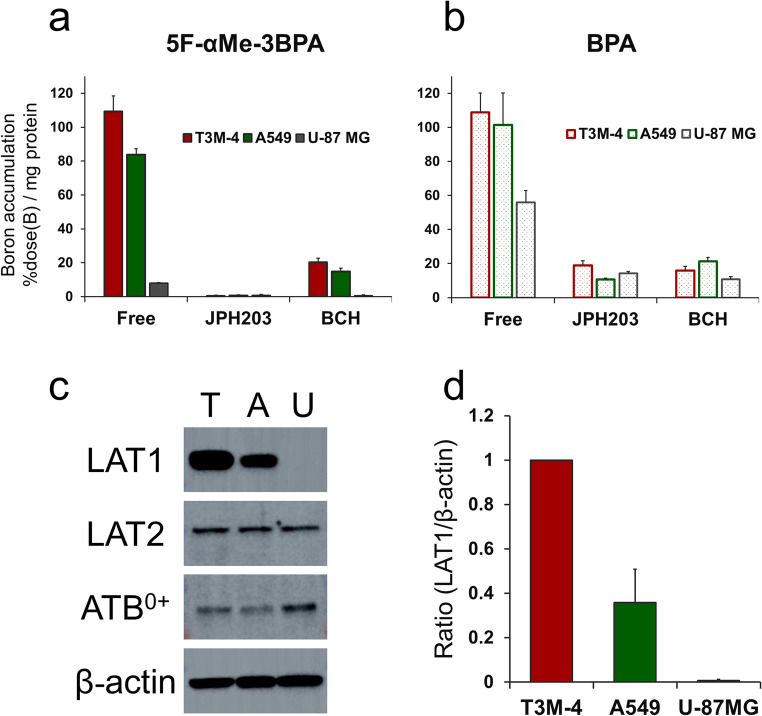
Boron uptake in cancer cell lines with varying LAT1 expression; (**a**, **b**) Boron accumulation after incubation with 5F-αMe-3BPA (**a**) and BPA (**b**) in T3M-4, A549, and U-87 MG cells under free uptake conditions and in the presence of inhibitors JPH203 (10 µM) and BCH (1 mM). Boron content was quantified by ICP-MS and expressed as percentage of added boron per mg cellular protein (%dose(B)/mg protein). (**c**) Western blot analysis of amino acid transporter expression in T3M-4 (T), A549 (A), and U-87 MG (U) cell lines. (**d**) Quantitative analysis of LAT1 protein expression normalized to β-actin levels. LAT1/β-actin ratios were calculated using ImageJ software from Western blot band intensities (*n* = 4)

The uptake of 5F-αMe-3BPA in T3M-4, A549, and U-87 MG cells was 109 ± 9, 84 ± 3, and 8 ± 0% dose(B)/mg protein, respectively (Fig. 2a). In comparison, BPA accumulation reached 109 ± 11, 101 ± 19, and 56 ± 7% dose(B)/mg protein in T3M-4, A549, and U-87 MG cells, respectively (Fig. 2b). Uptake of 5F-αMe-3BPA was almost completely suppressed by the LAT1-selective inhibitor JPH203. While BPA uptake was also reduced by JPH203, it remained notably higher than that of 5F-αMe-3BPA. Both compounds showed partial inhibition in the presence of the broad L-type amino acid transporter substrate BCH. Western blot analysis indicated relative LAT1 expression levels of 36 ± 15% in A549 and 0.7 ± 0.5% in U-87 MG cells, normalized to T3M-4 (100%). In contrast, LAT2 and ATB^0+^ expression was detected in U-87 MG cells (Fig. 2c and d). Full boron uptake data are presented in Table S1, and unprocessed Western blot images are shown in Figure S2.

### Tumor accumulation of 5F-αMe-3BPA in various LAT1 expressing models

**Fig. 3:**
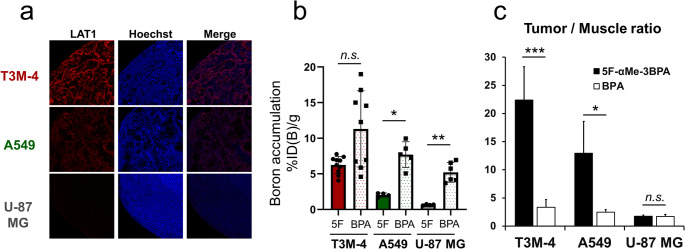
Boron accumulation in tumor xenograft models with varying LAT1 expression; (**a**) Immunofluorescence staining of LAT1 expression in tumor sections from T3M-4, A549, and U-87 MG xenografts. Tumor sections were stained with anti-LAT1 antibody. Cell nuclei were counterstained with Hoechst 33342. (**b**) In vivo boron accumulation in T3M-4, A549, and U-87 MG xenografts following intravenous administration of 5F-αMe-3BPA (5F) or BPA (%ID(B)/g). Differences in accumulation levels between 5F-αMe-3BPA and BPA are analyzed by Dunnett’s T3 multiple comparisons test (*p < 0.05, **p < 0.01; n.s. = not significant). (**c**) Tumor-to-muscle boron accumulation ratios for xenografts. Data were analyzed by Welch’s t-test (*p < 0.05, **p < 0.01, and ***p < 0.001)

Representative fluorescent images of LAT1 immunostaining in tumor sections are shown in Fig. 3a. LAT1 expression was strongest in T3M-4 tumors, moderate in A549, and nearly undetectable in U-87 MG. In vivo boron distribution following administration of 5F-αMe-3BPA or BPA in xenograft models is summarized in Fig. 3b, Figure S3, and Table S2. Tumor uptake of 5F-αMe-3BPA was 6.2 ± 1.2, 2.0 ± 0.3, and 0.7 ± 0.1%ID(B)/g in T3M-4, A549, and U-87 MG tumors, respectively, while BPA uptake reached 11 ± 5.3, 7.7 ± 1.8, and 5.2 ± 1.4%ID(B)/g. Considerable variability in T3M-4 tumors was observed with BPA treatment. Although no significant difference was noted between 5F-αMe-3BPA and BPA uptake in T3M-4 tumors, significant reductions were seen with 5F-αMe-3BPA in A549 and U-87 MG. Importantly, in normal tissues within the expected neutron irradiation field (muscle, skin, brain), boron levels were markedly lower with 5F-αMe-3BPA compared with BPA. For T3M-4 xenografts, the tumor-to-muscle (T/M) ratio was 22 ± 6 for 5F-αMe-3BPA, substantially exceeding the value of 3.4 ± 1.4 for BPA (Fig. 3c).

### Biodistribution study of 5-[¹⁸F]F-αMe-3BPA and [¹⁸F]FBPA

**Fig. 4:**
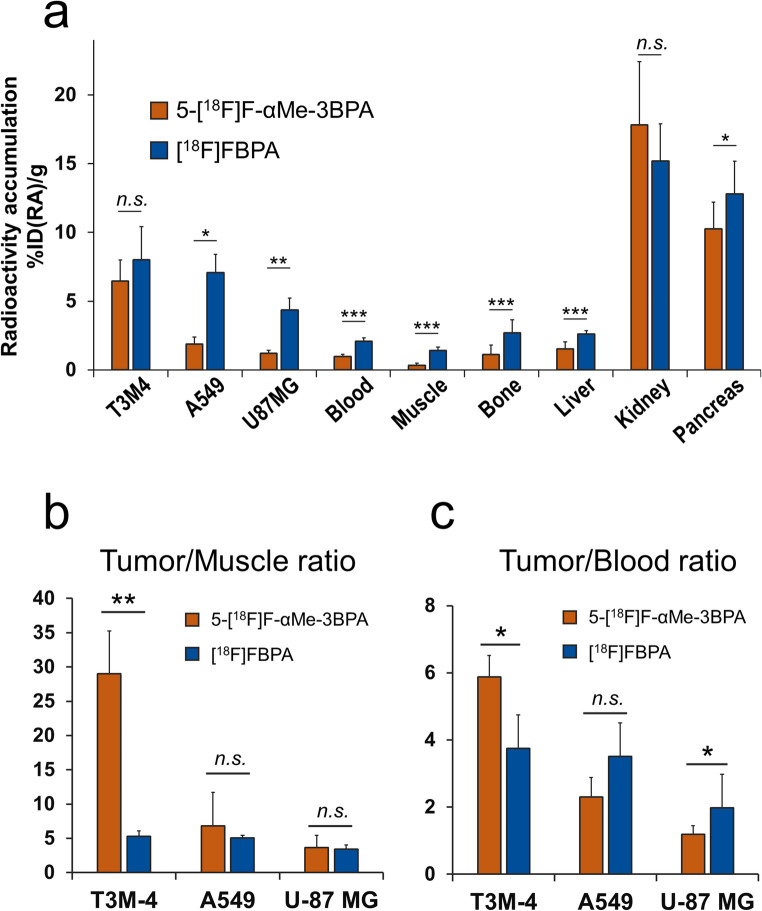
Comparison of 5-[¹⁸F]F-αMe-3BPA and [¹⁸F]FBPA biodistribution in xenograft tumor models (*n* = 4 per group); **(a)** Biodistribution of 5-[¹⁸F]F-αMe-3BPA and [¹⁸F]FBPA at 60 min after intravenous injection. Radioactivity accumulation is expressed as percentage of injected dose per gram of tissue (%ID(RA)/g). **(b)** Tumor-to-muscle, and **(c)** tumor-to-blood radioactivity ratios for 5-[¹⁸F]F-αMe-3BPA and [¹⁸F]FBPA. Data are analyzed by Welch’s t-test (*p < 0.05, **p < 0.01, and ***p < 0.001) compared with [¹⁸F]FBPA accumulation within corresponding tissues

5-[¹⁸F]F-αMe-3BPA was synthesized with a radiochemical conversion of 31 ± 7.4% (step1, maximum 41.5%, decay-corrected), an overall radiochemical yield of 5.3 ± 3.4% (maximum 11.6%, decay-corrected), and a radiochemical purity greater than 99% (*n* = 10). The total synthesis time from the initiation of the ¹⁸F-fluorination reaction was 114 ± 9 min. Radioactivity accumulation 60 min after administration of 5-[¹⁸F]F-αMe-3BPA and [¹⁸F]FBPA in tumor-bearing mice is shown in Fig. 4a, with detailed data provided in Table 1.

**Table 1:** Biodistribution of 5-[¹⁸F]F-αMe-3BPA and [¹⁸F]FBPA at 60 min after intravenous injection in xenograft tumor models (% injected dose per gram tissue (%ID(RA)/g))

Organ	5-[¹⁸F]F-αMe-3BPA	[¹⁸F]FBPA
T3M-4	6.5 ± 1.5	8.0 ± 2.4
A549	^*^1.9 ± 0.5	7.1 ± 1.3
U-87 MG	^**^1.2 ± 0.2	4.4 ± 0.9
Blood	^***^1.0 ± 0.2	2.1 ± 0.3
Muscle	^***^0.3 ± 0.2	1.4 ± 0.2
Skin	^***^0.6 ± 0.2	2.3 ± 0.8
Bone	^***^1.1 ± 0.7	2.7 ± 1.0
Brain	^***^0.1 ± 0.1	0.9 ± 0.2
Heart	^***^0.5 ± 0.1	1.8 ± 0.2
Lungs	^***^0.9 ± 0.2	1.9 ± 0.2
Liver	^***^1.5 ± 0.5	2.6 ± 0.3
Kidneys	17.8 ± 4.6	15.2 ± 2.7
Spleen	^***^1.0 ± 0.2	2.0 ± 0.2
Stomach^¶^	^***^0.3 ± 0.2	0.5 ± 0.1
Small intestine	^***^1.2 ± 0.2	1.9 ± 0.3
Large intestine	^***^0.5 ± 0.3	1.4 ± 0.2
Pancreas	^*^10.3 ± 1.9	12.8 ± 2.4
T3M-4/Blood	^*^5.9 ± 0.6	3.7 ± 1.2
A549/Blood	2.3 ± 0.6	3.5 ± 0.6
U-87 MG/Blood	^*^1.2 ± 0.3	2.0 ± 0.5
T3M-4/Muscle	^**^29.0 ± 6.2	5.3 ± 0.8
A549/Muscle	6.8 ± 4.9	5.1 ± 0.3
U-87 MG/Muscle	3.6 ± 1.8	3.4 ± 0.6

Data are presented as mean ± standard deviation and analyzed using Welch’s t-test; *p < 0.05, **p < 0.01, and ***p < 0.001 compared with the corresponding [¹⁸F]FBPA accumulation. Tumor data were obtained from each tumor type (*n* = 4), whereas normal tissue data were pooled from all tumor-bearing mice (total *n* = 12). One A549-bearing mouse in the [¹⁸F]FBPA group was excluded due to experimental failure (A549 tumors, *n* = 3 for [¹⁸F]FBPA). ^¶^Stomach values are expressed as % injected dose

Tumor uptake of 5-[¹⁸F]F-αMe-3BPA was 6.5 ± 1.5, 1.9 ± 0.5, and 1.2 ± 0.2%ID(RA)/g in T3M-4, A549, and U-87 MG tumors, respectively, compared with 8.0 ± 2.4, 7.1 ± 1.3, and 4.4 ± 0.9%ID(RA)/g for [¹⁸F]FBPA. 5-[¹⁸F]F-αMe-3BPA exhibited significantly lower accumulation in muscle and blood (0.3 ± 0.2 and 1.0 ± 0.2%ID(RA)/g, respectively) relative to [¹⁸F]FBPA (1.4 ± 0.2 and 2.1 ± 0.3%ID(RA)/g, respectively). In T3M-4 tumors, tumor-to-blood (T/B) and tumor-to-muscle (T/M) ratios for 5-[¹⁸F]F-αMe-3BPA were 5.9 ± 0.6 and 29 ± 6.2, respectively—both significantly higher than those observed for [¹⁸F]FBPA (T/B = 3.7 ± 1.2, T/M = 5.3 ± 0.8) (Fig. 4b). In contrast, the T/B ratio for 5-[¹⁸F]F-αMe-3BPA in U-87 MG tumors was 1.2 ± 0.3, significantly lower than that of [¹⁸F]FBPA (2.0 ± 0.5).

### Co-injection study of 5-[¹⁸F]F-αMe-3BPA/5F-αMe-3BPA and [¹⁸F]FBPA/BPA

**Fig. 5:**
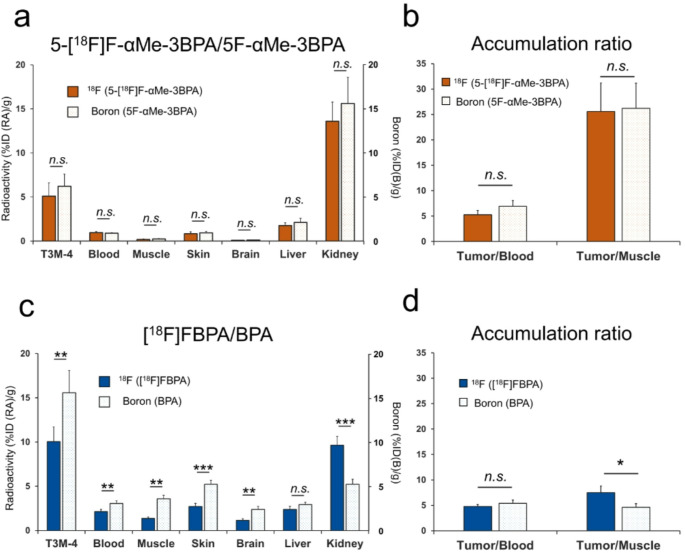
Co-injection studies comparing radioactivity and boron accumulation (*n* = 4 per group); (**a**, **c**) Biodistribution of co-injected 5F-αMe-3BPA/5-[¹⁸F]F-αMe-3BPA (**a**), and BPA/[¹⁸F]FBPA (**c**) in T3M-4 tumor-bearing mice 60 min post-injection. Dark color bars represent ¹⁸F radioactivity accumulation measured by gamma counter (%ID(RA)/g), whereas light color bars represent boron accumulation measured by ICP-MS (%ID(B)/g). (**b**, **d**) Tumor-to-blood and tumor-to-muscle ratios comparing radioactivity-based and boron-based measurements. Statistical significance between ¹⁸F and boron measurements were analyzed by Welch’s t-test (*p < 0.05, **p < 0.01, and ***p < 0.001)

Biodistribution data following co-injection of 5-[¹⁸F]F-αMe-3BPA (~ 0.3 pmol) with 5F-αMe-3BPA (5 µmol), or [¹⁸F]FBPA (~ 4 nmol) with BPA (5 µmol), are shown in Fig. 5a and c, with detailed results in Table S3. For 5-[¹⁸F]F-αMe-3BPA/5F-αMe-3BPA, no significant differences were observed between ¹⁸F accumulation (gamma counter) and boron accumulation (ICP-MS) across all organs. Conversely, for [¹⁸F]FBPA/BPA, ¹⁸F accumulation values were significantly lower than boron levels in T3M-4 tumors and most organs, except liver, kidneys, and bone. For 5-[¹⁸F]F-αMe-3BPA/5F-αMe-3BPA, T/M and T/B ratios derived from ¹⁸F and boron accumulation were comparable (Fig. 5b). In contrast, for [¹⁸F]FBPA/BPA, while the T/B ratio showed no significant difference, the T/M ratio based on radioactivity was significantly higher than that based on boron (Fig. 5d). Radio-TLC of urine samples at 1 h post-injection confirmed that more than 99% of the radioactivity corresponded to intact 5-[¹⁸F]F-αMe-3BPA (Fig. S4).

### PET imaging (5-[¹⁸F]F-αMe-3BPA and [¹⁸F]FBPA)

**Fig. 6:**
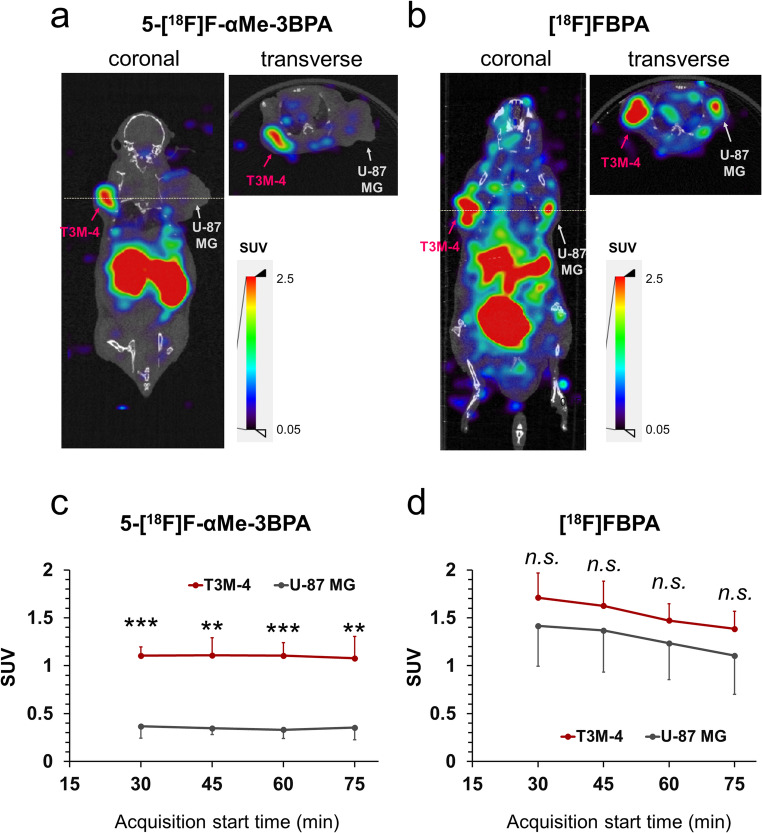
Representative PET/CT imaging of 5-[¹⁸F]F-αMe-3BPA and [¹⁸F]FBPA in dual tumor xenograft models (*n* = 4 per group); (**a**, **b**) Representative PET/CT images of 5-[¹⁸F]F-αMe-3BPA (**a**) and [¹⁸F]FBPA (**b**) obtained for 60–75 min post-intravenous injection in mice bearing bilateral T3M-4 and U-87 MG tumors. (**c**, **d**) Time-activity curves for 5-[¹⁸F]F-αMe-3BPA (**c**) and [¹⁸F]FBPA (**d**) showing SUV in T3M-4 and U-87 MG tumors. One T3M-4 tumor displaying extensive necrosis on [¹⁸F]FBPA PET imaging was excluded from the quantitative analysis. Data represent mean ± standard deviation. Statistical significance between SUV in T3M-4 and U-87 MG tumors were analyzed using paired t-tests (**p < 0.01, ***p < 0.001)

Representative PET images acquired 60–75 min after administration of 5-[¹⁸F]F-αMe-3BPA and [¹⁸F]FBPA in mice bearing T3M-4 (left) and U-87 MG (right) tumors are shown as CT-fused images in Fig. 6a and b, with corresponding MIP images provided in Fig.  S5. Temporal SUV changes following administration of 5-[¹⁸F]F-αMe-3BPA and [¹⁸F]FBPA are presented in Fig. 6c and d, respectively, and additional time point images are included in Fig. S6. PET with 5-[¹⁸F]F-αMe-3BPA enabled clear visualization of T3M-4 tumors, whereas U-87 MG tumors were not visually discernible (SUV: 1.1 ± 0.1 vs. 0.3 ± 0.1, respectively). In contrast, [¹⁸F]FBPA PET delineated both T3M-4 and U-87 MG tumors (SUV: 1.5 ± 0.2 vs. 1.2 ± 0.4, respectively). Comparative analysis of mean SUV values demonstrated distinct uptake profiles for 5-[¹⁸F]F-αMe-3BPA and [¹⁸F]FBPA. With 5-[¹⁸F]F-αMe-3BPA, T3M-4 tumors consistently showed significantly higher SUV values than U-87 MG tumors at all evaluated time points (Fig. 6c). In contrast, no significant SUV differences were observed between T3M-4 and U-87 MG tumors following [¹⁸F]FBPA administration (Fig. 6d).

## Discussion

In this study, we synthesized 5-[¹⁸F]F-αMe-3BPA, building on our previously identified LAT1-targeting compound [14], to assess its potential both as a LAT1 imaging probe and as part of the theranostic pair 5-[¹⁸F]F-αMe-3BPA/5F-αMe-3[¹⁰B]BPA. Optimal BNCT agents should exhibit high water solubility for high-dose administration, selective tumor accumulation, and the ability to reliably predict boron biodistribution [17, 18]. Our earlier work demonstrated that 5F-αMe-3BPA provides more than 15-fold higher LAT1/LAT2 selectivity and over 80-fold greater solubility compared with clinically used BPA [14]. This superior solubility eliminates the need for sugar complexation, thereby avoiding formulation-related issues such as acute kidney injury and hematuria [19, 20]. Furthermore, while BPA is transported not only via LAT1 but also to some extent by other transporters, including LAT2 and ATB⁰⁺ (SLC6A14) [6], it was indicated that 5F-αMe-3BPA shows minimal interaction with these alternative pathways, reducing nonspecific uptake in LAT1-negative normal tissues. This transporter selectivity enhances therapeutic specificity and strengthens its utility for pre-therapeutic LAT1 imaging. This study goes beyond our prior identification of 5F‑αMe‑3BPA [14] by creating the structure‑matched PET tracer 5‑[¹⁸F]F‑αMe‑3BPA and introduces three key advances: First, we validate LAT1-dependent uptake in human cancer cell lines with diverse transporter expression patterns, extending beyond the artificial overexpression model used previously. Second, we developed 5-[¹⁸F]F-αMe-3BPA as a highly LAT1-selective PET probe that achieves superior tumor-to-background contrast through minimal off-target transport, enabling accurate discrimination between LAT1-high and LAT1-low tumors—a capability not achieved with [¹⁸F]FBPA. Third, we establish that this structurally identical theranostic pair overcomes the pharmacokinetic mismatch inherent to the clinical [¹⁸F]FBPA/BPA combination, enabling accurate prediction of therapeutic boron biodistribution.

In vitro, boron accumulation of 5F-αMe-3BPA correlated closely with LAT1 expression levels across T3M-4, A549, and U-87 MG cells, and was completely inhibited by the LAT1-selective inhibitor JPH203. By contrast, BPA accumulation was only partially reduced by JPH203, consistent with contributions from LAT2 and ATB⁰⁺. This interpretation was further supported by the presence of LAT2 and ATB⁰⁺ proteins in U-87 MG cells, where BPA uptake persisted despite low LAT1 expression. Importantly, in vivo biodistribution results paralleled these in vitro observations: while 5F-αMe-3BPA achieved comparable tumor accumulation to BPA in LAT1-rich T3M-4 tumors, it demonstrated markedly lower uptake in normal tissues such as muscle, skin, and brain. Collectively, these findings indicate that BPA distribution reflects mixed transporter activity, whereas 5F-αMe-3BPA behaves predominantly as a LAT1 substrate in complex tumor environments, thereby offering superior tumor-to-background contrast.

For diagnostic use, we radiolabeled 5F-αMe-3BPA with ¹⁸F. Traditional clinical synthesis of [¹⁸F]FBPA relies on electrophilic substitution with F₂ gas [21, 22], whereas recent copper-catalyzed nucleophilic radiofluorination strategies [23–25] offer site-specific ¹⁸F incorporation, higher molar activity, and generally superior radiochemical yields. In our earlier assessment of fluorinated αMe-BPA derivatives, both 2F- and 5F-substituted analogues emerged as promising candidates due to favorable LAT1/LAT2 selectivity and high tumor-to-normal tissue ratios [14]. However, radiosynthesis of the ortho (2F) analogue requires di-Boc protection to suppress side reactions [26], whereas the meta (5F) analogue requires only mono-Boc protection, simplifying the radiosynthetic pathway. Based on these considerations, we selected the 5F scaffold as the optimal framework for establishing a structurally identical PET/BNCT theranostic pair. In line with this rationale, radiosynthesis of 5-[¹⁸F]F-αMe-3BPA was achieved with 31% radiochemical conversion, high radiochemical purity, and reasonable synthesis time, although the overall radiochemical yield was limited to 5% due to the three-step sequence. This radiosynthetic route demonstrates practical feasibility for preclinical use, and further optimization of the multi-step process should facilitate routine clinical production.

As a PET imaging probe, 5-[¹⁸F]F-αMe-3BPA provided high in vivo contrast, driven primarily by its minimal uptake in LAT1-negative tissues such as muscle, highlighting the benefit of reduced off-target transport. This led to a markedly improved T/M ratio and enabled clear discrimination between LAT1-high and LAT1-low tumors—an outcome not achieved with [¹⁸F]FBPA. Compared with other α-methyl amino acid-based LAT1 tracers, the advantage of 5-[¹⁸F]F-αMe-3BPA is also striking: while [¹⁸F]FAMT [27] and [¹⁸F]FAMP [28] typically achieve T/M ratios of ~ 4, 5-[¹⁸F]F-αMe-3BPA reached values nearly an order of magnitude higher. These features support its role not only as a theranostic partner for BNCT but also as a stand-alone LAT1 imaging probe. Given that LAT1 is an emerging therapeutic target for small-molecule inhibitors [29] and TAT [30], the ability to noninvasively quantify LAT1 expression has potential utility across diverse therapeutic settings.

A fundamental requirement for PET/BNCT theranostics is that the PET tracer faithfully reflects the biodistribution of the therapeutic agent. Our experimental results demonstrate that 5F-αMe-3BPA exhibits sufficient in vivo stability, with negligible bone uptake and urinary radioactivity predominantly as the intact compound. In the conventional [¹⁸F]FBPA/BPA system, [¹⁸F]FBPA exhibits improved LAT1/LAT2 selectivity compared to BPA due to the addition of ¹⁸F [31], and consequently has a larger molecular size. Furthermore, as we demonstrated in our previous study, introduction of fluorine into phenylalanine derivatives alters lipophilicity (log P) [14]. These combined changes contribute to biodistribution discrepancies between the diagnostic and therapeutic agents. In contrast, our 5-[¹⁸F]F-αMe-3BPA/5F-αMe-3BPA theranostic pair maintains identical LAT1/LAT2 selectivity and physicochemical properties. Therefore, co-injection experiments confirmed near-identical distributions of ¹⁸F and boron for the 5-[¹⁸F]F-αMe-3BPA/5F-αMe-3BPA pair. Such concordance is crucial for reliable treatment planning and dosimetry in BNCT—a limitation that has long restricted the clinical utility of the [¹⁸F]FBPA/BPA combination. By overcoming this issue, the 5-[¹⁸F]F-αMe-3BPA/5F-αMe-3BPA pair enables PET imaging to function as a direct surrogate for therapeutic boron delivery.

For BNCT, the favorable biodistribution profile of 5F-αMe-3BPA has important clinical implications. Its reduced accumulation in normal tissues such as muscle, skin, and brain suggests that higher neutron doses can be delivered to LAT1-positive tumors without exceeding normal tissue tolerance—an obstacle that currently limits BPA-based BNCT [32, 33]. This expanded therapeutic window may enhance nuclear reaction efficiency and enable more selective eradication of LAT1-positive tumor cells while minimizing collateral damage to surrounding tissues. Critically, 5-[¹⁸F]F-αMe-3BPA PET also permits pre-therapeutic assessment of LAT1 expression, supporting patient selection and individualized planning for 5F-αMe-3[¹⁰B]BPA BNCT.

A common challenge in PET/BNCT theranostics is the potential mismatch between tracer-level and therapeutic-level dosing, which can result in differences in biodistribution [34]. In current BNCT protocols, boron agents are administered by prolonged continuous infusion rather than by bolus injection, which may also lead to different pharmacokinetic behavior. Although LAT1-mediated transport is generally less prone to saturation than receptor–ligand systems, partial saturation has been reported under high-dose BNCT conditions [6]. In our study, high-dose administration of 5F-αMe-3BPA led to reduced tumor uptake in LAT1-low U-87 MG tumors compared with tracer-level 5-[¹⁸F]F-αMe-3BPA, consistent with competitive inhibition of residual LAT1 transport. Such tumors are intrinsically unsuitable for LAT1-targeted BNCT, and this effect is unlikely to limit clinical translation. Moving forward, it will be important to assess whether 5-[¹⁸F]F-αMe-3BPA PET can quantitatively predict therapeutic boron distribution under clinical dosing conditions of 5F-αMe-3[¹⁰B]BPA, while accounting for potential LAT1 saturation. In particular, longitudinal evaluation of tracer kinetics is warranted, as the current study examined only a single time point. Dynamic PET combined with kinetic modeling could enable estimation of transporter capacity, providing a more reliable basis for dose prediction than static SUV analysis alone.

In conclusion, we demonstrated that 5F-αMe-3BPA displays high LAT1 selectivity and that 5-[¹⁸F]F-αMe-3BPA offers outstanding tumor-to-background contrast while accurately mirroring the biodistribution of the therapeutic compound. By overcoming key limitations of the BPA/[¹⁸F]FBPA pair, this structurally identical theranostic combination establishes a pharmacokinetically aligned platform for BNCT. Furthermore, the excellent imaging performance of 5-[¹⁸F]F-αMe-3BPA underscores its potential as a stand-alone LAT1 PET tracer, supporting broader applications in the development and clinical implementation of LAT1-targeted therapies.

## Supplementary Information

Below is the link to the electronic supplementary material.

Supplementary Material 1 (5.06 MB)

## Data Availability

Data are available from the corresponding author on reasonable request.
