# Supplementary material for: A LAT1-selective PET tracer, 5-[¹⁸F]F-αMe-3BPA, as a companion to its structurally matched ¹⁰B analog in boron neutron capture therapy
Source: Eur J Nucl Med Mol Imaging. 2025 Nov 18;53(4):2566–77. doi: 10.1007/s00259-025-07668-3 (PMC12920373; doi:10.1007/s00259-025-07668-3)
Supplement: Supplementary file 1 — Supplementary Material 1 (5.06 MB) [file 259_2025_7668_MOESM1_ESM.docx]

**Supplementary Information for:**

A LAT1-selective PET tracer, 5-[^18^F]F-αMe-3BPA, as a companion to its structurally matched ^10^B analog in Boron Neutron Capture Therapy

N. Kondo *et al.*


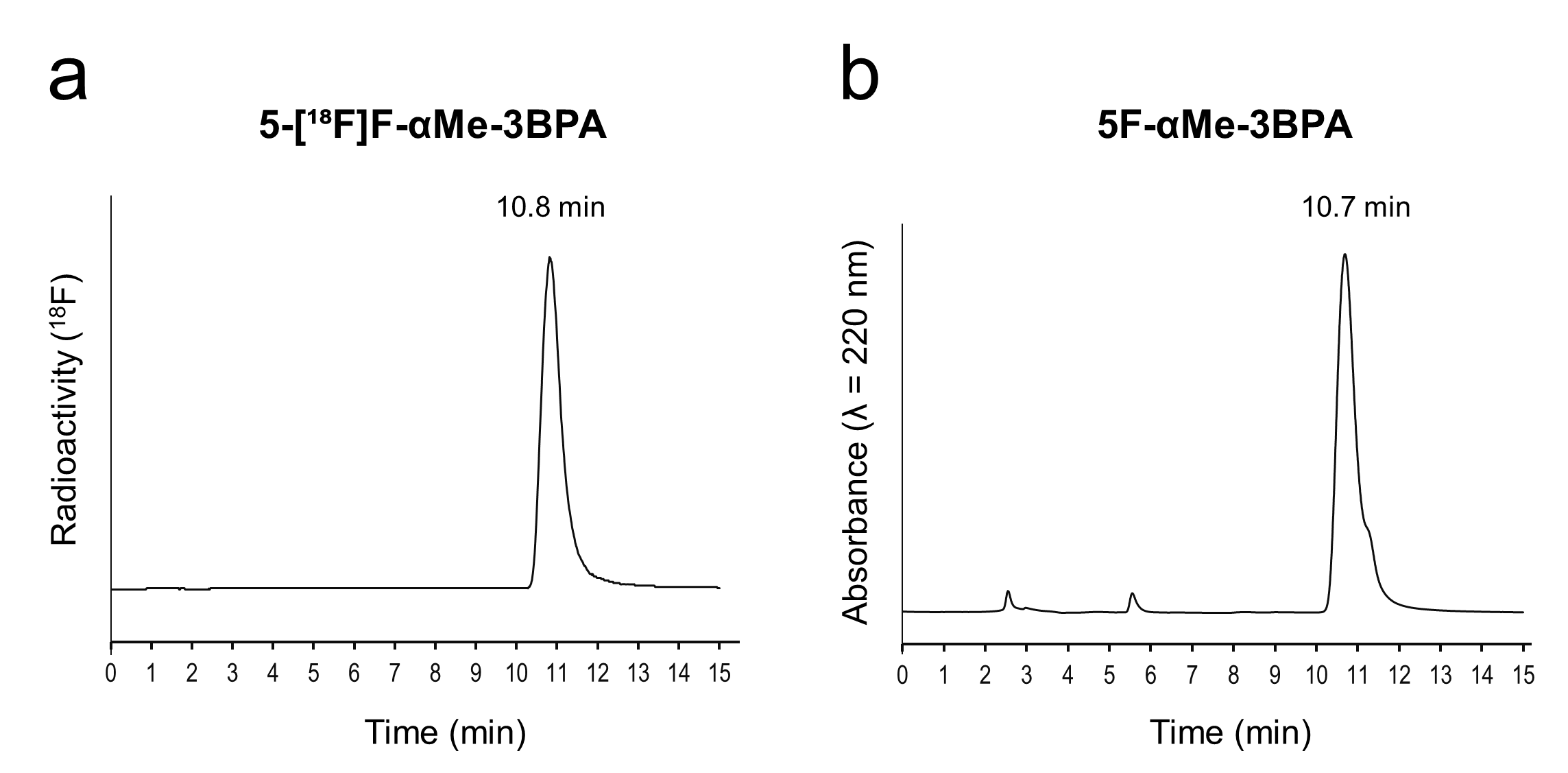


**Fig. S1**

Analytical co-elution HPLC chromatograms of purified 5-[^18^F]F-αMe-3BPA and non-radiolabeled 5F-αMe-3BPA. (a) Radioactive detection of purified 5-[^18^F]F-αMe-3BPA (retention time = 10.8 min). (b) UV absorbance detection (λ = 220 nm) of non-radiolabeled 5F-αMe-3BPA standard (retention time = 10.7 min).


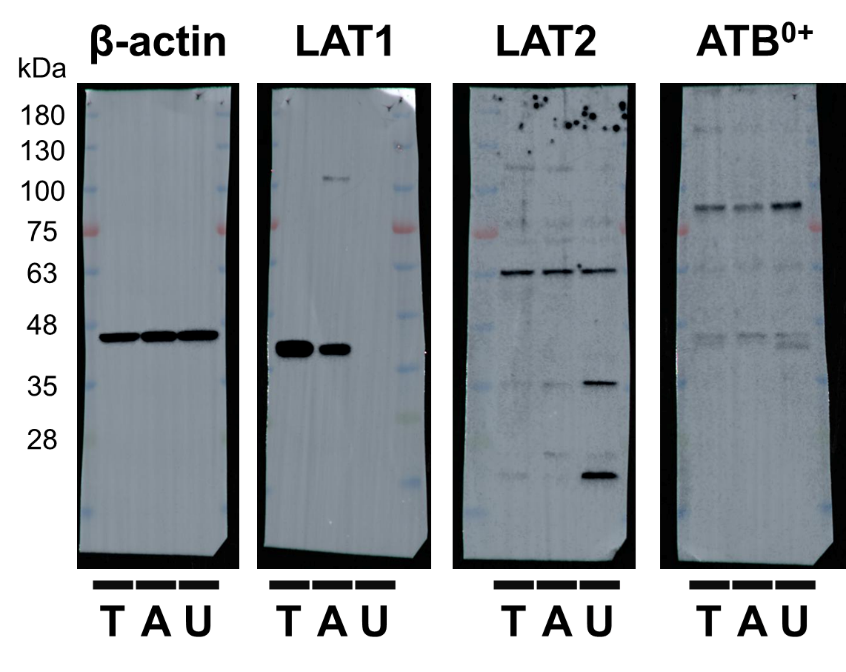


**Fig. S2**

Unprocessed Western blot images of amino acid transporter expression in T3M-4 (T), A549 (A), and U-87 MG (U) cell lines with FastGene Bluestar prestained protein marker.


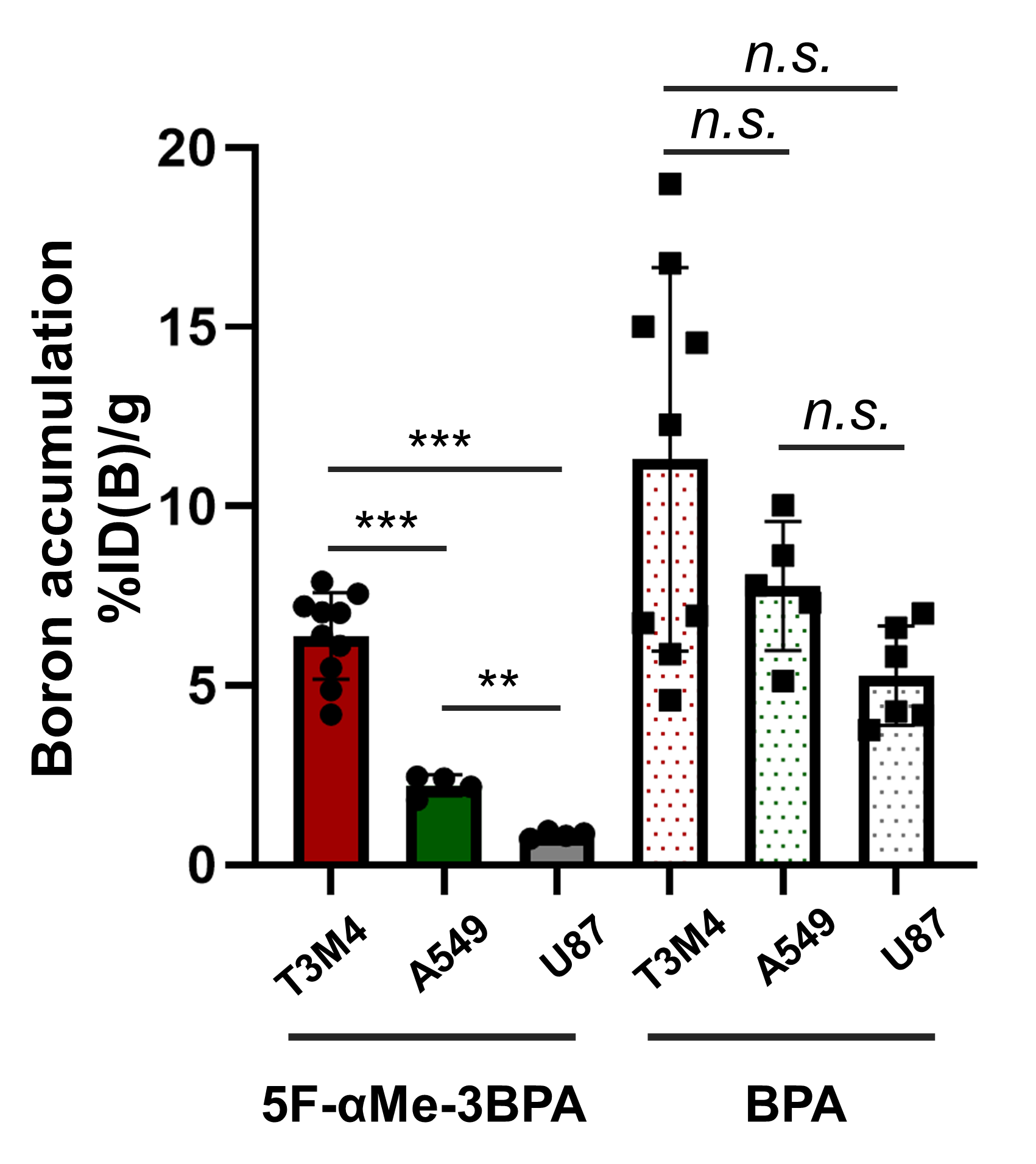


**Fig. S3**

Comparison of in vivo boron accumulation in T3M-4, A549, and U-87 MG xenografts following intravenous administration of 5F-αMe-3BPA or BPA (%ID(B)/g). Differences in accumulation levels between xenografts are analyzed by Dunnett's T3 multiple comparisons test; **p < 0.01, ***p < 0.001.


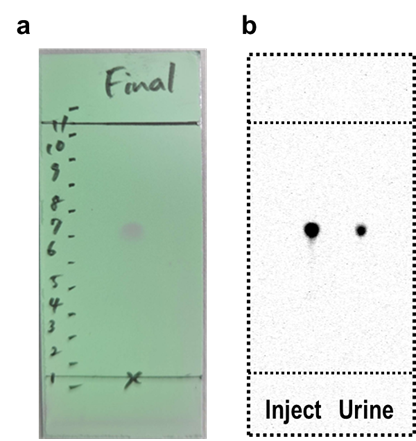


**Fig. S4**

TLC analysis of 5-[^18^F]F-αMe-3BPA metabolic stability in urine.

(a) TLC plate showing UV visualization (254 nm) of non-radioactive 5F-αMe-3BPA standard compound. The plate was developed using n-BuOH/water/acetic acid (12:5:3, v/v/v) as mobile phase (Rf = 0.63) (b) Autoradiographic analysis comparing radioactivity distribution between injection solution (Inject) and urine sample (Urine) collected 60 min post-intravenous administration of 5-[^18^F]F-αMe-3BPA (5.0 MBq/100 μL) into mice.


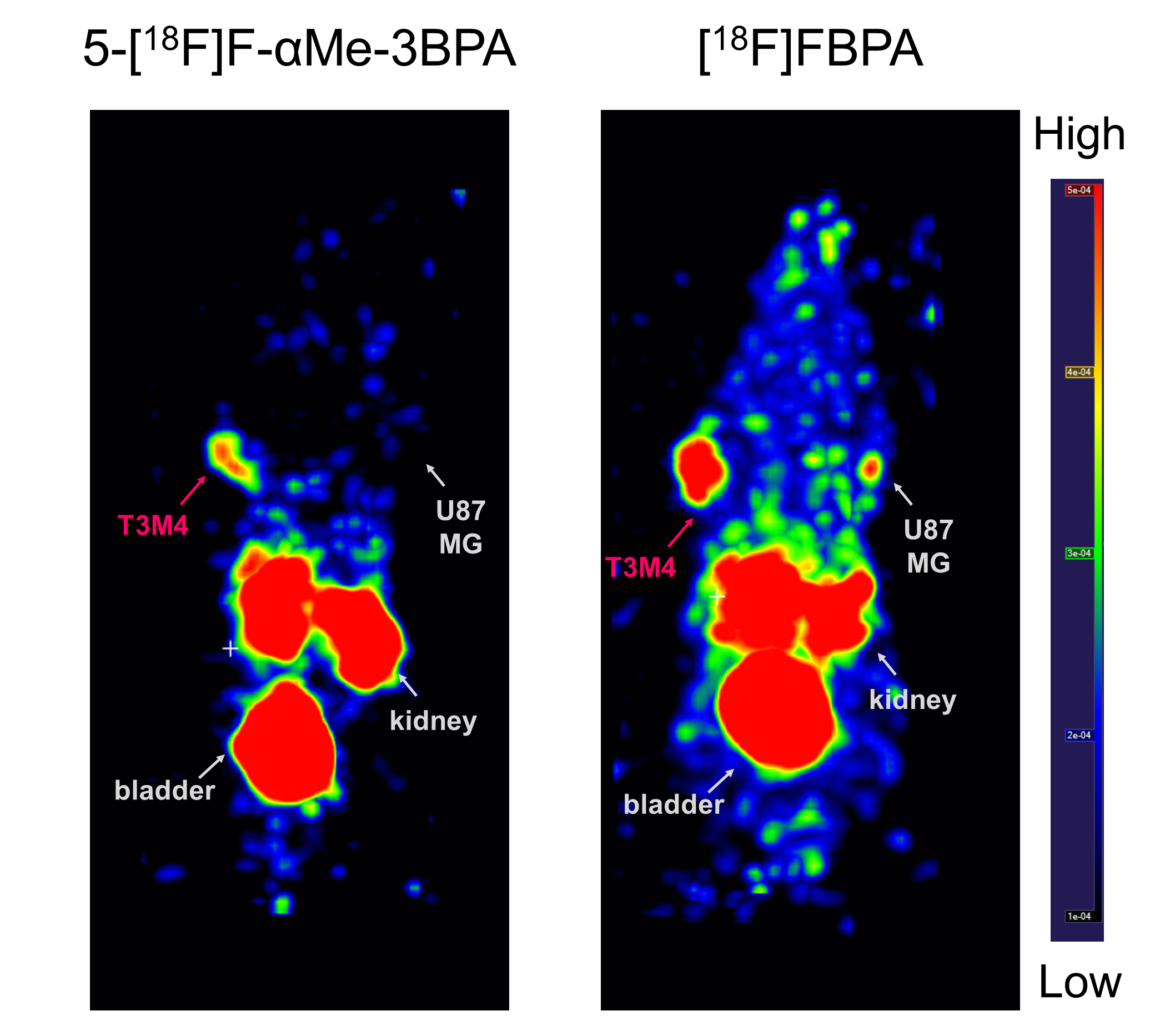


**Fig. S5**

Maximum intensity projection PET images of Fig. 6 (left; 5-[^18^F]F-αMe-3BPA, right; [^18^F]FBPA) at 60 min post-intravenous injection in mice bearing bilateral T3M-4 and U-87 MG tumors.


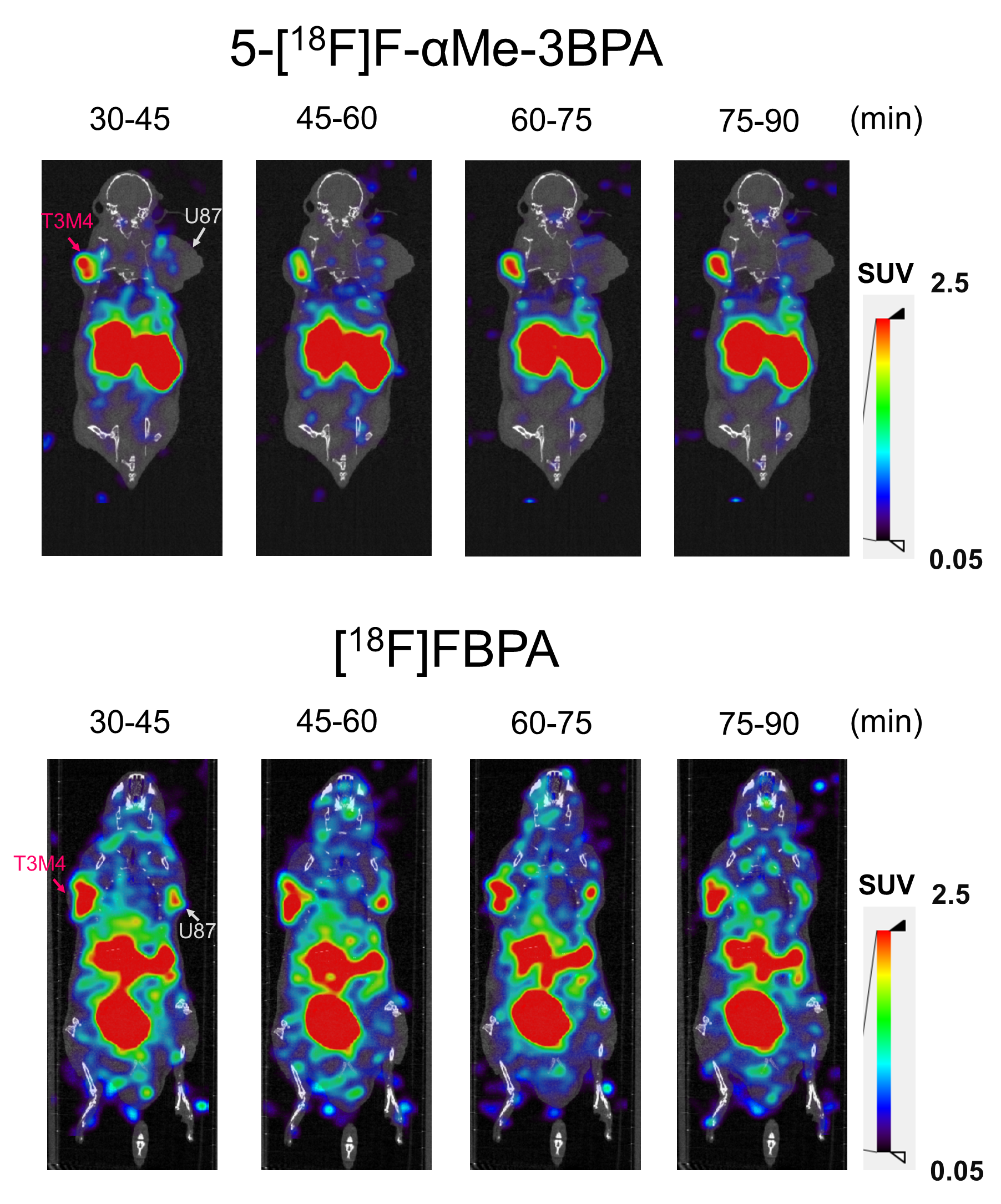


**Fig. S6**

Sequential PET/CT images of 5-[^18^F]F-αMe-3BPA (upper panel) and [^18^F]FBPA (lower panel) at multiple time points (30-45, 45-60, 60-75, and 75-90 min) post-intravenous injection in mice bearing bilateral T3M-4 and U-87 MG tumors.

**Fig. S7** ^1^H-NMR spectrum of compound 3

**Fig. S8** ^13^C-NMR spectrum of compound 3

**Fig. S9** ^1^H-NMR spectrum of compound 4

**Fig. S10** ^13^C-NMR spectrum of compound 4

**Table S1**

Boron uptake in cancer cells 30 min after incubation with 5F-αMe-3BPA and BPA (% added boron dose per mg protein, n = 3)

|  |  | Free | With JPH203 | With BCH |
| --- | --- | --- | --- | --- |
| 5F-αMe-  3BPA | T3M-4 | 109.3 ± 9.1 | 0.4 ± 0.5 | 20.3 ± 2.4 |
|  | A549 | 83.8 ± 3.4 | 0.6 ± 0.5 | 14.8 ± 1.9 |
|  | U-87 MG | 7.9 ± 0.3 | 0.8 ± 0.6 | 0.5 ± 0.7 |
| BPA | T3M-4 | 108.8 ± 11.4 | 19.0 ± 2.7 | 16.0 ± 2.4 |
|  | A549 | 101.4 ± 18.9 | 10.6 ± 0.8 | 21.2 ± 2.3 |
|  | U-87 MG | 56.0 ± 6.9 | 14.2 ± 1.0 | 10.8 ± 1.5 |

**Table S2**

Biodistribution of boron after administration of 5F-αMe-3BPA and BPA in tumor-bearing mice 60 min post-injection (% injected dose per gram tissue (%ID(B)/g))

| Organ | 5F-αMe-3BPA (n = 18) | BPA (n = 20) |
| --- | --- | --- |
| T3M-4 | 6.2 ± 1.2 (n = 10) | 11.3 ± 5.3 (n = 9) |
| A549 | 2.0 ± 0.3 (n = 4) | 7.7 ± 1.8 (n = 5) |
| U-87 MG | 0.7 ± 0.1 (n = 4) | 5.2 ± 1.4 (n = 6) |
| Blood | 1.0 ± 0.2 | 2.7 ± 0.5 |
| Plasma | 0.7 ± 0.2 | 2.1 ± 0.3 |
| Muscle | 0.3 ± 0.1^§^ | 3.2 ± 0.4 |
| Skin | 1.0 ± 0.4 | 4.1 ± 1.2 |
| Bone | 1.0 ± 0.7 | 1.7 ± 0.5 |
| Brain | 0.1 ± 0.1 | 1.7 ± 0.5 |
| Heart | 0.5 ± 0.2 | 3.2 ± 0.6 |
| Lungs | 1.2 ± 0.3 | 3.0 ± 0.5 |
| Liver | 1.7 ± 0.5 | 2.3 ± 0.5 |
| Kidneys | 13.3 ± 3.4 | 5.3 ± 0.8 |
| Spleen | 1.4 ± 0.4 | 3.7 ± 0.7 |
| Pancreas | 11.8 ± 3.8 | 22.2 ± 7.4 |

Data are expressed as mean ± standard deviation

For 5F-αMe-3BPA, tumor tissue data were obtained separately for each tumor type—T3M-4 (n = 10), A549 (n = 4), and U‑87 MG (n = 4), whereas normal tissue data were pooled across all tumor-bearing mice (n = 18).

For 5F-αMe-3BPA, tumor tissue data were obtained separately for each tumor type—T3M-4 (n = 9), A549 (n = 5), and U‑87 MG (n = 6), whereas normal tissue data were pooled across all tumor-bearing mice (n = 20).

^§^Three samples at the ICP-MS detection limit were excluded from the analysis.

**Table S3**

Biodistribution of co-injected 5-[^18^F]F-αMe-3BPA/5F-αMe-3BPA and [^18^F]FBPA/BPA in T3M-4-bearing mice 60 min post-injection (n = 4 per group, % injected dose per gram tissue (%ID/g))

|  | 5-[^18^F]F-αMe-3BPA/5F-αMe-3BPA | | [^18^F]FBPA/BPA | |
| --- | --- | --- | --- | --- |
| Organ | Radioactivity  (5-[^18^F]F-αMe-3BPA) | Boron  (5F-αMe-3BPA) | Radioactivity  ([^18^F]FBPA) | Boron  (BPA) |
| T3M-4 | 5.1 ± 1.5 | 6.2 ± 1.4 | ^**^10.6 ± 1.3 | 16.3 ± 2.0 |
| Blood | 1.0 ± 0.1 | 0.9 ± 0.1 | ^**^2.2 ± 0.2 | 3.0 ± 0.3 |
| Muscle | 0.2 ± 0.0 | 0.2 ± 0.1 | ^**^1.4 ± 0.1 | 3.6 ± 0.4 |
| Skin | 0.9 ± 0.2 | 0.9 ± 0.2 | ^***^2.7 ± 0.4 | 5.1 ± 0.5 |
| Bone | 0.9 ± 0.2 | 0.4 ± 0.4 | 2.8 ± 0.5 | 2.1 ± 0.4 |
| Brain | 0.1 ± 0.0 | 0.1 ± 0.0 | ^**^1.2 ± 0.2 | 2.4 ± 0.3 |
| Heart | 0.4 ± 0.1 | 0.4 ± 0.1 | ^**^2.2 ± 0.3 | 3.4 ± 0.5 |
| Lungs | 0.9 ± 0.2 | 1.1 ± 0.2 | ^**^2.0 ± 0.4 | 3.2 ± 0.4 |
| Liver | 1.8 ± 0.3 | 2.1 ± 0.5 | 2.5 ± 0.2 | 2.9 ± 0.2 |
| Kidneys | 13.6 ± 2.2 | 15.6 ± 3.0 | ^***^10.0 ± 0.7 | 5.2 ± 2.6 |
| Spleen | 1.2 ± 0.3 | 1.6 ± 0.5 | ^**^2.1 ± 0.4 | 4.1 ± 0.6 |
| Pancreas | 11.2 ± 3.4 | 13.6 ± 3.9 | ^**^17.1 ± 4.1 | 33.0 ± 4.6 |
| T3M-4/Blood | 5.3 ± 0.9 | 6.9 ± 1.1 | 4.8 ± 0.4 | 5.4 ± 0.6 |
| T3M-4/Muscle | 25.6 ± 5.6 | 26.2 ± 5.0 | *7.5 ± 1.3 | 4.6 ± 0.7 |

Data are expressed as mean ± standard deviation and analyzed by Welch’s t-test;

*p < 0.05, **p < 0.01, and ***p < 0.001 compared to the corresponding accumulation of BPA

Supplementary Material and Methods

**Precursor Synthesis**

Scheme 1. Synthesis of 5-[^18^F]F-αMe-3BPA precursor.

*Reagent and conditions*: (a) *tert*-butyl 2-((4-chlorobenzylidene)amino) propanoate, (*R*)-Maruoka cat., 80% CsOH aq., toluene, -5 °C; (b) (i) citric acid, THF/H_2_O, rt; (ii) Boc_2_O, Na_2_CO_3_, MeCN/H_2_O, rt; (c) bis(pinacolato)diboron, AcOK, Pd(PPh_3_)_2_Cl_2_, DMSO, 100 °C.

*Step 1*. To a mixture of *tert*-butyl 2-((4-chlorobenzylidene)amino) propanoate (3.78 g, 14.1 mmol), 1-bromo-3-(bromomethyl)-5-iodobenzene (6.32 g, 16.8 mmol), and (*R*)-4,4-dibutyl-2,6-bis(3,4,5-trifluorophenyl)- 4,5-dihydro-3H-dinaphtho[2,1-*c*:1′,2′-*e*]azepinium bromide (10.9 mg, 14.6 μmol) in toluene (30 mL) was added dropwise an 80% cesium hydroxide (25 g) aqueous solution at below -5 °C. The mixture was stirred at -5 °C for 2 days, after which the reaction mixture was poured into water and extracted with toluene. The organic layer was washed with brine, dried over anhydrous sodium sulfate, and concentrated under reduced pressure to produce crude **2**.

*Step 2*. Crude **2** was dissolved in tetrahydrofuran (26 mL), citric acid (26.9 g, 140 mmol) in water (80 mL) was added thereto, and the mixture was stirred at room temperature for 3 h. The reaction mixture was subjected to distillation under reduced pressure to remove tetrahydrofuran. The residue was washed with ethyl acetate, after which the aqueous layer was adjusted to at least pH 8 using potassium carbonate. The aqueous layer was then extracted with ethyl acetate. The organic layer was washed with brine, dried over anhydrous sodium sulfate, and concentrated under reduced pressure. The resulting residue was purified by silica gel column chromatography (hexane/ethyl acetate) to yield crude *tert*-butyl (S)-2-amino-3-(3-bromo-5-iodophenyl)-2-methylpropanoate (3.07 g) as a yellow oil.

*Step 3*. Crude *tert*-butyl (S)-2-amino-3-(3-bromo-5-iodophenyl)-2-methylpropanoate (3.07 g) in acetonitrile (28 mL) was added sodium carbonate (1.45 g, 13.7 mmol) in water (15 mL) and di-*tert*-butyl dicarbonate (1.90 mL, 8.27 mmol). The reaction mixture was stirred at room temperature for 10 h. Subsequently, acetonitrile was removed by distillation under reduced pressure, and the resulting aqueous layer was extracted with ethyl acetate. The organic layer was washed with brine, dried over anhydrous sodium sulfate, and concentrated under reduced pressure. The residue was purified by silica gel column chromatography (hexane/ethyl acetate) to yield **3** (2.67 g, 35% in 3 steps) as a white solid.

^1^H NMR (Fig. S7, 400 MHz, CDCl_3_): *δ* 7.71 (m, 1H), 7.41 (m, 1H), 7.25 (m, 1H), 5.33 (brs, 1H), 3.44 (d, *J* = 13.6 Hz, 1H), 3.06 (d, *J* = 13.6 Hz, 1H), 1.57 (s, 3H), 1.51 (s, 9H), 1.50 (s, 9H). ^13^C NMR (Fig. S8, 100 MHz, CDCl_3_): *δ* 172.4, 154.2, 141.2, 137.8, 137.6, 132.5, 122.5, 94.0, 82.9, 79.5, 60.5, 39.7, 28.5, 28.0, 24.2. HRMS (EI) *m*/*z* calcd for C_19_H_27_BrINO_4_ [M]^+^ 539.0168; found, 539.0169.

*Step 4*. A mixture of **3** (2.67 g, 4.94 mmol), bis(pinacolato)diboron (1.51 g, 5.95 mmol), bis(triphenylphosphine)palladium(II) dichloride (0.17 g, 0.242 mmol), potassium acetate (0.73 g, 7.44 mmol), and DMSO (22 mL) was stirred at 100 °C for 6 h under nitrogen atmosphere. The reaction mixture was cooled, poured into water and extracted with ethyl acetate. The organic layer was washed with brine, dried over anhydrous sodium sulfate, and concentrated under reduced pressure. The residue was purified by silica gel column chromatography (hexane/ ethyl acetate) to yield **4** (2.14 g, 80%) as a white solid.

^1^H NMR (Fig. S9, 400 MHz, CDCl_3_): *δ* 7.78 (m, 1H), 7.49 (s, 1H), 7.38 (m, 1H), 5.33 (brs, 1H), 3.49 (d, *J* = 13.6 Hz, 1H), 3.10 (d, *J* = 13.6 Hz, 1H), 1.59 (s, 3H), 1.50 (s, 18H), 1.31 (d, *J* = 4.0 Hz, 12H). ^13^C NMR (Fig. S10, 100 MHz, CDCl_3_): *δ* 172.7, 154.2, 138.7, 135.9, 135.7, 135.0, 122.1, 84.0, 82.6, 79.2, 60.5, 40.2, 28.5, 27.9, 25.0, 24.8, 24.2. HRMS (EI) *m*/*z* calcd for C_25_H_39_BBrNO_6_ [M]^+^ 539.2054; found, 539.2062.

**Radiochemical conversion (RCC) determination**

RCC was determined by normal-phase thin-layer chromatography (TLC) using EtOAc:hexane (1:8, v/v) as the mobile phase. The TLC plate was developed for 4 cm, then sectioned into 4 mm segments, and radioactivity was measured using a gamma counter. RCC was calculated as the ratio of radioactivity in the product-containing fractions to total radioactivity.

**Western Blotting**

Cultured cells were lysed in Passive Lysis Buffer (Promega, Madison, WI, USA), homogenized via sonication, and centrifuged to remove cellular debris. The resulting supernatant was subsequently diluted in a sample buffer (70 mM Tris, 1% SDS, 11% glycerol, 0.005% bromophenol blue, 10% 2-mercaptoethanol). Aliquots of the samples (10 µL, 1.0 mg/mL) were subjected to gel electrophoresis, followed by Western blot analysis. Primary antibodies utilized included anti-LAT1 (KE026, 0.1 µg/mL, Medicinal Chemistry Pharmaceutical, Sapporo, Japan), anti-LAT2 (HPA051950, 0.2 µg/mL, Sigma-Aldrich Co. LLC, St. Louis, MO, USA), and anti-ATB^0+^ (BMP052, 1:1000 dilution, Medical & Biological Laboratories, Tokyo, Japan). A horseradish peroxidase-conjugated monoclonal antibody (1:3000 dilution, HAF008, R&D Systems, Minneapolis, MN, USA) served as the secondary antibody.

β-Actin levels were quantified using an anti-β-actin antibody (1:5000 dilution, NB600-503, Novus Biologicals, Littleton, CO, USA) as a loading control for protein quantification. Immunoreactive bands were visualized using Chemi-Lumi One L (Nacalai Tesque). The FastGene Bluestar prestained protein marker (NIPPON Genetics Co, Ltd, Tokyo, Japan) was employed as a molecular weight standard. Band visualization was performed using the Amersham Imager 600 system (GE Healthcare Japan, Tokyo, Japan). Quantification of the immunoreactive bands was conducted using ImageJ software. The expression levels of LAT1 in tumor cells were normalized to β-actin levels and expressed as the LAT1/β-actin ratio (n = 3).

**Cellular uptake study**

T3M-4 cells were seeded in 12-well plates two days before the experiment. Culture medium was removed, and cells were washed twice with Hank's balanced salt solution (HBSS: 125 mM sodium chloride, 25 mM HEPES, 4.8 mM potassium chloride, 5.6 mM D-glucose, 1.3 mM calcium chloride, 1.2 mM magnesium sulfate, and 1.2 mM potassium dihydrogen phosphate, pH 7.4). Cells were then incubated with HBSS (450 μL) with or without inhibitor (10 μM JPH203 or 1 mM BCH) at 37°C for 5 min. Subsequently, 1 mM 5F-αMe-3BPA or BPA complexed with D-fructose (0.11 w/v%) (50 μL) was added (final conc. 100 μM) and incubated at 37°C for 30 minutes. After washing twice with HBSS, cells were lysed with 0.2 M NaOH (400 μL). Cellular protein content was measured using BCA protein assay kits. Cells were ashed with nitric acid, and boron content was quantified using an 8800 triple quadrupole inductively coupled plasma mass spectrometry (ICP-MS). Boron accumulation in cells was calculated as the percentage of added boron per mg of cell protein (%dose(B)/mg protein).

**Immunohistochemistry (excised tumor)**

Tumors were excised from T3M-4, A549, and U-87 MG tumor-bearing mice, embedded in O.C.T. Compound (Sakura Finetek Japan Co., Ltd., Tokyo, Japan), and immediately frozen at -80°C. The frozen tumors were sectioned into 10 μm-thick slices using a microtome (Shiraimatsu Co., Ltd., Osaka, Japan). The frozen sections were fixed with acetone at -20°C, washed with phosphate-buffered saline containing 0.1% Tween 20 (PBS-T_20_), and blocked with Blocking One Histo for 10 minutes. Anti-LAT1 antibody was added and incubated overnight at 4°C. After washing with PBS-T_20_, anti-rabbit IgG (H + L), CF555 (2.5 μg/mL, SAB4600068, Sigma-Aldrich Co. LLC) was added and incubated at room temperature for 45 minutes. Following another wash with PBS-T_20_, Hoechst 33342 (5 μg/mL) was added and incubated at room temperature for 10 minutes. After washing with water, fluorescence images were captured using a BZ-X810 fluorescence microscope (Keyence Co., Osaka, Japan).

**Urinary metabolite analysis**

T3M-4 tumor-bearing mice were intravenously injected with 5-[^18^F]F-αMe-3BPA (5.0 MBq/100 μL). Urine was collected directly from bladder 60 min post-injection. After protein precipitation with acetonitrile, samples were filtered through GL Chromato Disk 13P (0.45 μm, GL Sciences, Tokyo, Japan). TLC was carried out using n-BuOH/water/acetic acid (12/5/3) as mobile phase. Plates were then exposed to an imaging plate (BAS-SR, Fujifilm, Tokyo, Japan) for 60 min, and radioactivity distribution was visualized with an Amersham Typhoon scanner (Cytiva, Tokyo, Japan).
